# Microbiological profiles of Gram-negative bacteria isolated from respiratory samples of mechanically ventilated patients at a tertiary Moroccan hospital

**DOI:** 10.1016/j.ijregi.2026.100969

**Published:** 2026-08-11

**Authors:** Sobha El Ftouh, Houssain Tligui, Jordi Vila, Larbi Aberouch, Tarek Dendane, Latifa Oualili, Oussama Ssouni, Mamoun Faroudy, Amine Ali Zeggwagh, Mouhssine Doumiri, Cristina Pitart, Maria Piquet, Elisabet Guiral, Khalid Abidi

**Affiliations:** 1 Center for Doctoral Studies in Life and Health Sciences, Faculty of Medicine and Pharmacy of Rabat, Mohammed V University in Rabat, Rabat, Morocco; 2 Research Laboratory of Ibn Sina University Hospital Centre, Faculty of Medicine and Pharmacy of Rabat, Mohammed V University in Rabat, Rabat, Morocco; 3 Department of Clinical Microbiology, Hospital Clínic, School of Medicine, ISGlobal, Barcelona, Spain; 4 Centro de Investigación Biomédica en Red (CIBER) de Enfermedades Infecciosas (CIBERINFEC), Instituto Salud Carlos III, Madrid, Spain; 5 Anesthesiology and Critical Care, Ibn Sina University Hospital, Faculty of Medicine and Pharmacy of Rabat, Mohammed V University in Rabat, Rabat, Morocco; 6 Medical Intensive Care Unit, Ibn Sina University Hospital, Faculty of Medicine and Pharmacy of Rabat, Mohammed V University in Rabat, Rabat, Morocco; 7 Department of Anaesthesiology and Intensive Care Medicine, National Institute of Oncology, Rabat, Faculty of Medicine and Pharmacy of Rabat, Mohammed V University in Rabat, Rabat, Morocco; 8 Department of Neurocritical Care, Neurocritical Care Unit, Rabat Specialty Hospital, Faculty of Medicine and Pharmacy of Rabat, Mohammed V University in Rabat, Rabat, Morocco; 9 Barcelona Institute for Global Health (ISGlobal), Hospital Clinic, Barcelona, Spain; 10 School of Medicine and Health Sciences, University of Barcelona, Barcelona, Spain; 11 Laboratory of Biostatistics, Clinical, and Epidemiological Research, Faculty of Medicine and Pharmacy of Rabat, Mohammed V University, Rabat, Morocco

**Keywords:** FT-IR, PFGE, VAP, ICU, MDR, Morocco

## Abstract

**Objectives:**

To characterise the Gram-negative bacteria implicated in ventilator-associated pneumonia (VAP), focusing on their antimicrobial resistance profiles and phylogenetic relationships.

**Methods:**

Seventy–eight isolates were collected from protected distal samples over two years; patients were under mechanical ventilation in the three adult ICUs of a Moroccan tertiary hospital. Isolates were identified using VITEK2. PFGE and FTIR determined genetic relationships, whereas the detection of genetic resistance determinants was carried out using PCR.

**Results:**

Carbapenem-resistant *Acinetobacter baumannii* harbouring the *bla*-_OXA-23_ was the predominant Gram-negative bacterium. *Klebsiella pneumoniae* was the most prevalent among the Enterobacterales, with a high resistance rate to almost all antibiotics, due to the coexistence of (*bla*) _CTX-M-1, TEM, SHV_, and _OXA-48-like_ genes. *Escherichia coli* exhibited high resistance to ciprofloxacin (86%), ceftazidime (86%), and cotrimoxazole (71%). *Proteus mirabilis* was highly resistant to cotrimoxazole (60%) and ticarcillin (80%). *Pseudomonas aeruginosa* was resistant to carbapenems in 39% of cases, in which *bla*_NDM_ and *bla*_VIM_ were the only metallo-beta-lactamase genes detected. In phylogenetic analysis, both FTIR and PFGE techniques underscored no epidemiological relationship among strains.

**Conclusion:**

The prevalence of MDR and XDR bacteria among VAP cases in a tertiary hospital in Morocco is increasing, potentially due to the coexistence of antimicrobial resistance genes.

## Highlights

• Oxacillinase-23-producing *Acinetobacter baumannii* was the main non-fermenter in ventilator-associated pneumonia cases.
• *Klebsiella pneumoniae* predominated among enterobacterial species, co-harboring extended-spectrum β-lactamase and carbapenemase genes.
• Pulsed-field gel electrophoresis and Fourier-transform infrared showed strong clustering concordance in *Proteus mirabilis* (adjusted Rand index = 1).
• Epidemiological profiling showed high genetic heterogeneity across isolates.

## Introduction

Mechanical ventilation is a vital intervention that saves many lives in intensive care units (ICUs). Unfortunately, it is associated with infections such as ventilator-associated pneumonia (VAP) [1]. VAP is linked to increased morbidity, a prolonged duration of mechanical ventilation, and higher mortality rates in the ICU [2]. In low- and middle-income countries, the rates ranged from 10 to 41.7/1000 mechanical ventilation days [3].

Although numerous studies have reported high resistance rates in the ICU setting [4], there are few data related to VAP. The most common bacteria causing VAP worldwide are

*Acinetobacter baumannii* ranging from 4% to 50% [5]*, Pseudomonas aeruginosa* showing rates from 11% to 46% [6], *Klebsiella pneumoniae* varying from 8-56%, and *Escherichia coli* ranging from 10% to 22% [4,6]. Concerning *Stenotrophomonas maltophilia,* the range is 0.3%-2.0% [7]. These bacteria are becoming multidrug-resistant (MDR) worldwide [8], underscoring the global rise of extended-spectrum β-lactamase (ESBL)-producing *K. pneumoniae, E. coli*, and carbapenem-resistant *A. baumannii* [9]. This underscores the urgent need for updated data on VAP and its associated bacterial pathogens.

In Morocco, a nationwide survey exploring VAP-related infections across different tertiary hospitals was reported, showing proportions of extensively drug-resistant (XDR) bacteria, such as 78% *Acinetobacter* sp*.* and 24% Enterobacterales. However, neither the mechanisms of resistance nor the epidemiological relationships among the isolates were investigated [10]. This study, conducted in the capital city of Morocco, aimed to characterize Gram-negative bacteria associated with ventilator-associated infections, focusing on their antimicrobial resistance profiles, underlying mechanisms of resistance, and phylogenetic relationships.

## Materials and methods

### Study design

We conducted a prospective observational study involving 173 patients aged 18 years or older who were hospitalized in the Medical, Surgical, or Neurological ICUs and receiving mechanical ventilation at Ibn Sina University Hospital. We included all patients under mechanical ventilation for more than 48 h and excluded those with chest trauma or thoracotomy. The study was carried from July 2022 to January 2024.

### Sample collection

Once the protected distal sampling was performed, the samples were immediately transported to the Research Laboratory of Ibn Sina University Hospital for processing. The procedures included quantitative microbiological culture and Gram staining. Protected distal samples were promptly cultured on blood agar, Gram-positive cocci-selective medium, chocolate agar, and MacConkey agar. The threshold for culture positivity was ≥10³ colony-forming units/mL.

### Bacterial isolates

Clinical isolates were identified using the VITEK®2 compact system (bioMérieux, Marcy-l’Étoile, France). Antimicrobial resistance profiles were also generated using the same system according to the European Committee on Antimicrobial Susceptibility Testing interpretative criteria (clinical breakpoints 2022, version 1.0, May). The isolates were then stored at -80°C in the research laboratory at Ibn Sina Hospital in Rabat for subsequent analysis by the Antimicrobial Resistance Research Group at the Barcelona Institute for Global Health (ISGlobal). Seventy-eight isolates were confirmed at the species level using matrix-assisted laser desorption/ionization time-of-flight mass spectrometry (Bruker Daltonik GmbH, Bremen, Germany) at the Antimicrobial Resistance Research Centre of ISGlobal. Then, based on the pulsed-field gel electrophoresis (PFGE) dendrograms, only 73 isolates were genotyped by Fourier-transform infrared (FT-IR) spectroscopy (Figure 1). Phenotypic detection of carbapenemases was performed using the rapid test NG-Test-CARBA5 (NG.Biotech Laboratoires) (including KPC, NDM, VIM, OXA-48-like, and IMP), and the rapid test NG-Test-OXA-23 (NG.Biotech Laboratoires). Phenotypic detection of β-lactamases was performed using the rapid test NG-Test-CTX-M-Multiple (NG.Biotech Laboratoires). We then explored the CTX-M type by conventional polymerase chain reaction (PCR). Further PCR was carried out for other antimicrobial resistance genes such as *bla*_TEM_, *bla*_SHV_, *bla*_VIM_, *bla*_NDM_, tet(A), and *tet(B).*

**Figure 1:**
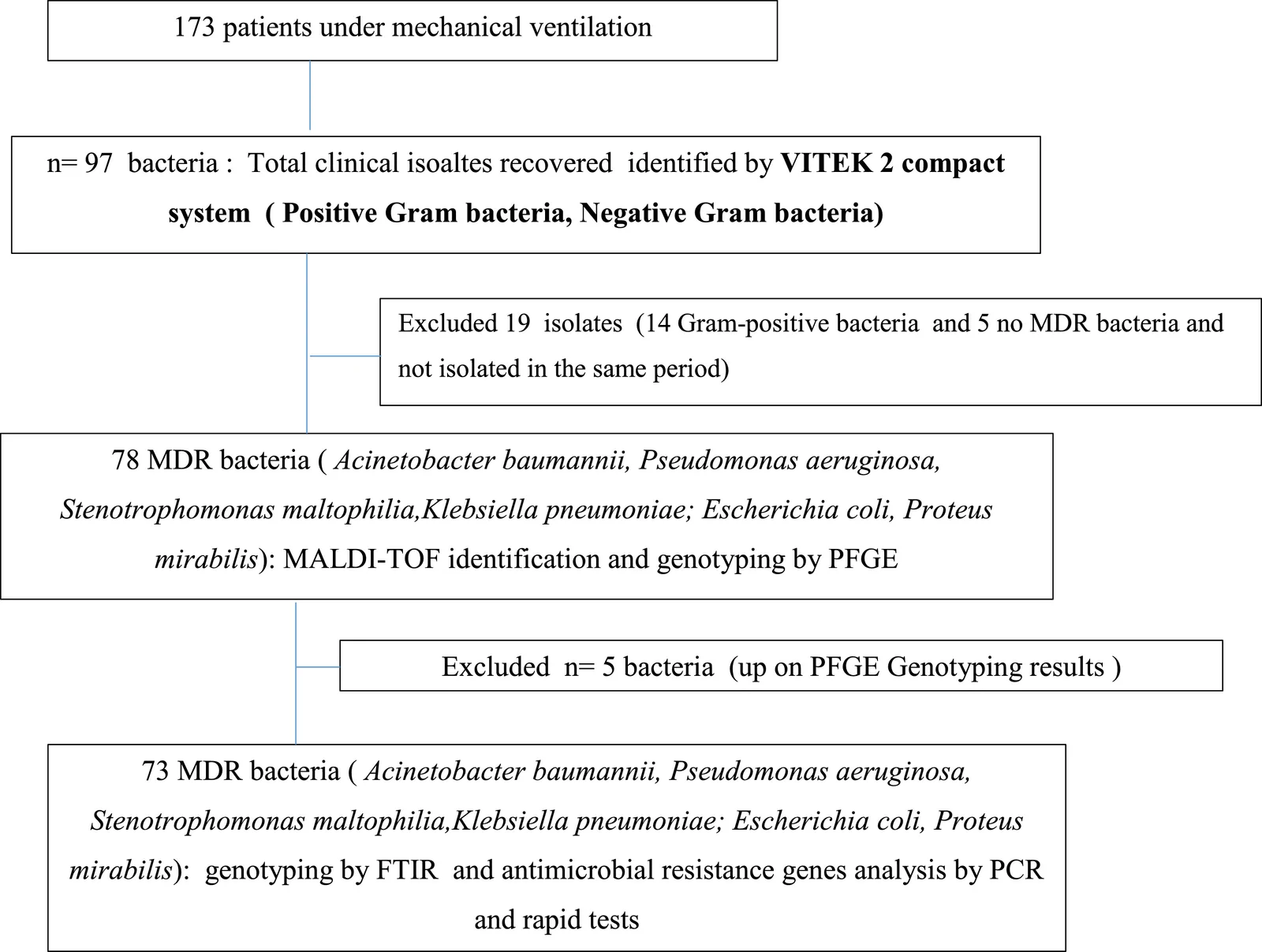
Workflow diagram for isolate selection and analysis. Clinical isolates from 173 ICU patients were identified using the VITEK2 Compact System. After excluding non-MDR and Gram-positive isolates, 78 Gram-negative MDR isolates were confirmed by MALDI-TOF and genotyped by PFGE. Based on the PFGE results, 73 isolates were selected for FT-IR genotyping, PCR detection of resistance genes, and rapid resistance testing. FT-IR, Fourier-transform infrared spectroscopy; ICU, intensive care unit; MDR, multidrug-resistant; MALDI-TOF, matrix-assisted laser desorption/ionization time-of-flight mass spectrometry; PCR, polymerase chain reaction; PFGE, pulsed-field gel electrophoresis.

### Molecular genotyping

The genetic relationships among the isolates were assessed using PFGE with the CHEF DR III and CHEF MAPPER systems from Bio-Rad. This process involved DNA restriction using specific enzymes tailored for each species. We used the NotI enzyme for *Proteus*, ApaI for *Acinetobacter*, SpeI for *Pseudomonas*, and XbaI for Enterobacterales other than *Proteus* and *S. maltophilia.* Dendrograms were created using InfoQuest software version 4.5, employing Dice similarity coefficients with arithmetic averaging to characterize relationships among PFGE profiles. Isolates with a Dice similarity index of 80% or higher were grouped into the same PFGE cluster.

### Sample preparation for FT-IR spectroscopy analysis

Bacterial isolates (*A. baumannii, P. aeruginosa, K. pneumoniae, E. coli, and P. mirabilis),* which exhibited similar pulsotypes and resistance genes, were first cultured on Tryptic Soy Agar (Trypticase Soy Agar) for 24 h at 37°C. After incubation, loopfuls of these isolates were suspended in 65 µL of sterile water. The suspensions were vigorously homogenized using small sterile metal cylinders in Eppendorf tubes before 65 µL of 70% ethanol was added. The suspensions were then homogenized at high speed. A 15-µL aliquot of each suspension was placed in five replicates on a silicon plate and dried for 5 min at 37°C. A total of six FT-IR silicon plates were analyzed over three different days. Each sample was examined five times, and the spectral averages were calculated for each isolate, as well as the American Type Culture Collection strain. The cut-off values were 0.209 for *A. baumannii*, 0.234 for *K. pneumoniae*, 0.312 for *P. aeruginosa, 0.051 for E. coli, and 0.111 for P. mirabilis*.

### PCR detection of extended-spectrum β-lactamases and carbapenemases

DNA was extracted from freshly cultured bacterial isolates by suspending colonies in 100 µL of sterile ultrapure water, heating at 96°C for 10 min, and subsequently incubating at 4°C. The primer profiles of *bla*_CTX-M_, *bla*_TEM_*, bla*_SHV_*, bla*_VIM_*, bla*_NDM, tet(A),_ and tet(B), as well as the thermocycling conditions, are detailed in Table 1S in the Supplementary Material.

### Data analysis

A general overview of all qualitative variables was provided. We used Excel software version 2017 to create tables and graphs. For clustering concordance, we used the Simpson index of diversity to evaluate the discriminatory power of the two typing methods. The concordance between PFGE and FT-IR was calculated using the adjusted Rand index. We used an online tool (www.comparingpartitions.info) to analyze the quantitative data regarding the discriminatory power and concordance of the typing methods.

## Results

### Antimicrobial resistance

Ninety-seven bacterial isolates were recovered from 173 patients on mechanical ventilation, including Gram-negative and Gram-positive bacteria. Of these, 78 isolates were selected for further analysis at the microbiology research laboratory in Barcelona to describe their epidemiology in mechanically ventilated patients (Figure 1). The selection criteria for the 78 isolates were based on Gram type, antimicrobial susceptibility testing results (particularly for MDR isolates), and, when possible, on isolates from the same ICU that recurred during the same period, even if they were antibiotic-susceptible. Then, based on PFGE genotyping profiles, 73 isolates were genotyped with FT-IR. They included five isolates of *P. mirabilis*, seven of *E. coli*, 18 of *K. pneumoniae*, 26 of *A. baumannii*, 17 of *P. aeruginosa,* and two of *S. maltophilia.* The three Enterobacterales, *E. coli, K. pneumoniae, and P. mirabilis,* showed high resistance to aminopenicillins, quinolones (ciprofloxacin), and cotrimoxazole (Figure 2).

**Figure 2:**
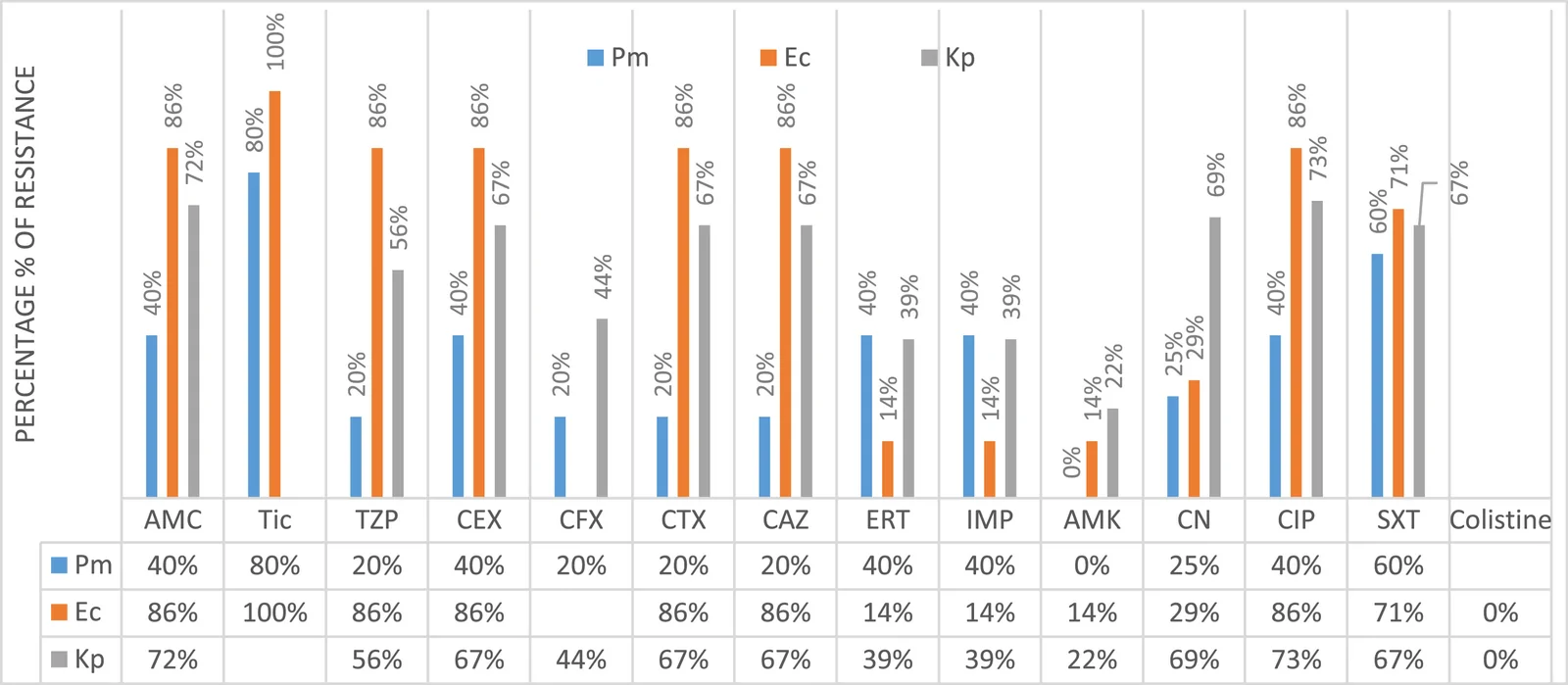
Antibiotic resistance rates of the three main Enterobacterales to different antibiotics. Each color on the bar corresponds to a bacterial type and indicates the percentage of resistance to the antimicrobials listed in the table. Bacteria: Ec, *Escherichia coli*; Kp, *Klebsiella pneumoniae*; Pm, *Proteus mirabilis*. Antibiotics: AMC, clavulanic acid-amoxicillin; AMK, amikacin; CAZ, ceftazidime; CEX, cefalexn; CFX, cefoxitin; CIP, ciprofloxacin; CN, gentamicin; CTX, cefotaxime; ERT, ertapenem; IMP, imipenem; SXT, trimethoprim/sulfamethoxazole; Tic, ticarcillin; TZP, piperacillin-tazobactam.

*P. mirabilis* isolates were highly resistant to ticarcillin (80%) and cotrimoxazole (60%). The detailed antimicrobial susceptibility, gene distribution, and resistance levels are presented in Figure 2, Figure 3, Figure 4.

*E. coli* showed high susceptibility to tigecycline, colistin, and fosfomycin (7/7, 100%) (Figure 2). Isolates characterized as ESBL and ESBL-like (5/7, 71%) carried the *bla_CTX-M-1_* gene, which confers resistance to third-generation cephalosporins (ceftazidime, cefotaxime, 86%). One *E. coli* isolate harbored only the *bla_TEM_* gene (1/7). Additionally, one isolate was resistant to carbapenems and harbored two carbapenemase genes, *bla*_OXA-48-like_ *and bla*_NDM,_ along with the *bla*_TEM_ gene (14%, 1/7) (Figure 3). These are associated with reduced antibiotic susceptibility. Eighty-six percent of *E. coli* isolates were described as MDR (Figure 4).

**Figure 3:**
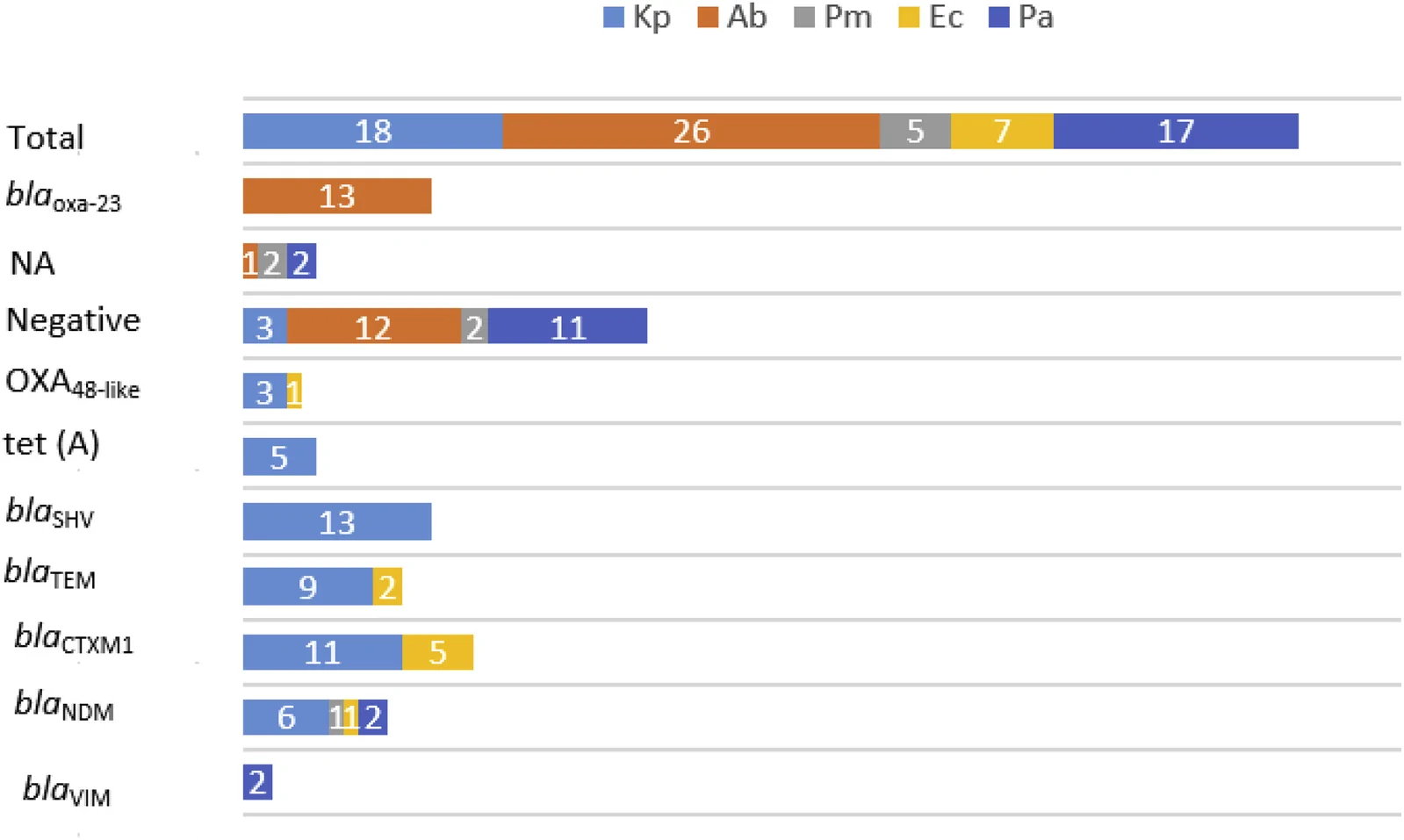
Gene distribution for the five species of Gram-negative bacteria. Each color on the bar corresponds to a bacterial type and indicates the type and the number of resistance genes detected in each species. Tot, the total number of antimicrobial resistance genes by bacterium. Gram-negative bacteria: Ab, *Acinetobacter baumannii*; Ec, *Escherichia coli*; Kp, *Klebsiella pneumoniae*; Pa, *Pseudomonas aeruginosa*; Pm, *Proteus mirabilis*. Antimicrobial resistance genes: CTX-M, cefotaximase-Munich; NDM, New Delhi metallo-β-lactamase; OXA-23, oxacillinase-23; OXA-48-like, oxacillinase-48; SHV, sulfhydryl variable beta-lactamase gene; TEM, temoniera beta-lactamase gene; TET(A), tetracycline resistance protein, class A; VIM, verona integron-encoded metallo-β-lactamase.

**Figure 4:**
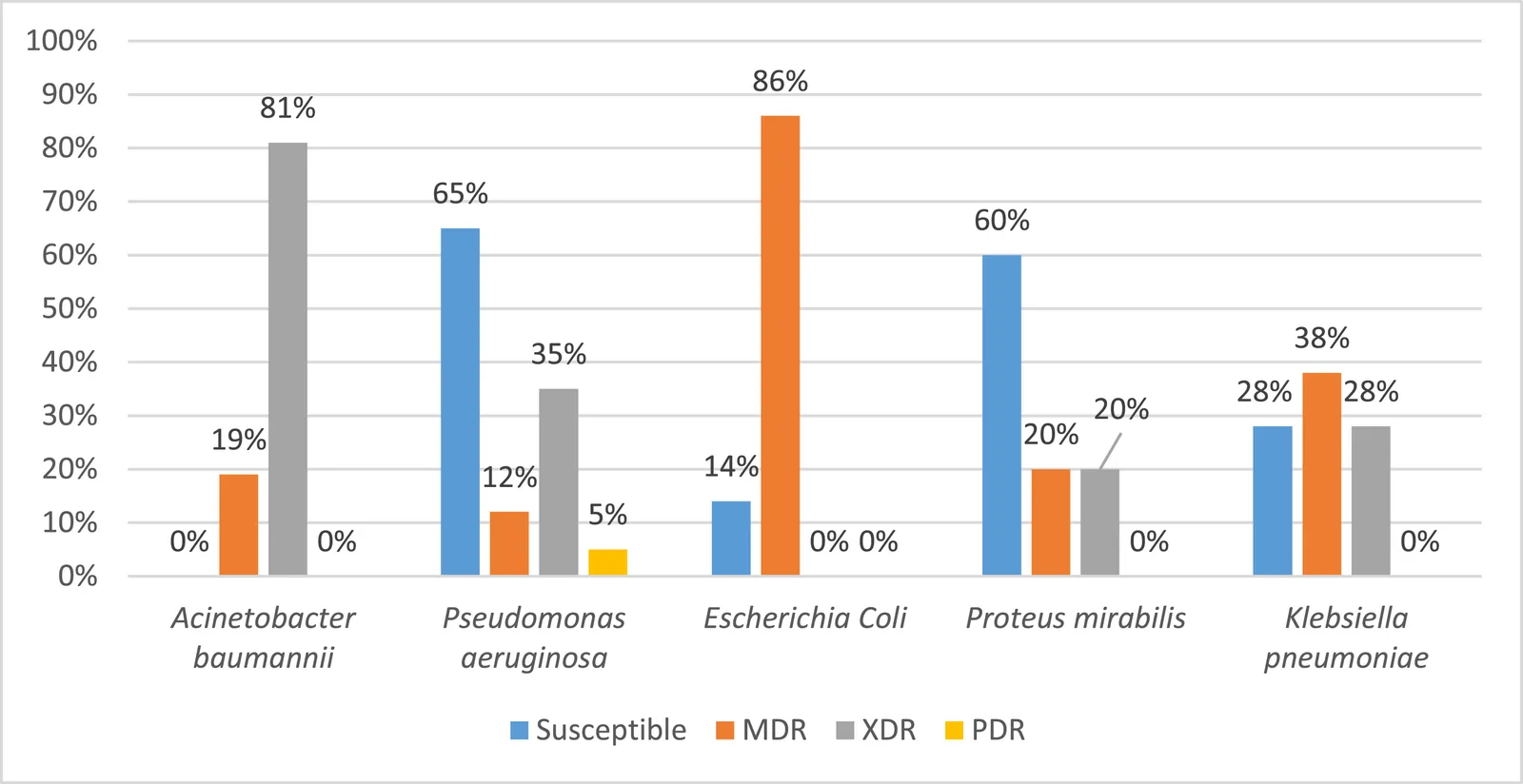
Resistance levels (MDR, XDR, PDR) by bacterium. Each color on the bar corresponds to the percentage of resistance levels (Susceptible, MDR, XDR, PDR) for each bacterium. MDR, multidrug-resistant; PDR, pandrug resistant; XDR, extensively drug resistant.

*K. pneumoniae* was more resistant than *E. coli* to carbapenems (imipenem, 39% vs 14%) (Figure 2, Figure 4). *K. pneumoniae* uniquely co-harbored numerous resistance genes. They included *bla*_CTX-M-1_ 11/18 *(61*%*), bla*_TEM_ 9/18 *(*50%)*, bla*_SHV_ 13/18 *(72*%*), bla*_NDM_ 6/18 *(33*%*), bla*_OXA-48-like_ 3/18 *(11*%*),* and tet(A) 5/18 (27%). These genes were found in different combinations**.** Eleven of the 18 isolates (61%) were genotypically ESBL-positive, carrying the *bla*_CTX-M-1_ gene. Phenotypically, four isolates expressed a non-masked ESBL phenotype, while the remaining seven co-carried carbapenemase genes that masked ESBL expression; they included four co-harboring *bla*_NDM_, *bla*_CTX-M-1_*, bla*_TEM,_ and *bla*_SHV_, and two showing a combination of *bla*_OXA-48-like,_ *bla*_CTX-M-1_, *bla*_TEM_**,** and *bla*_SHV_. One isolate carried *bla*_OXA-48-like_*, bla*_NDM_*, bla*_CTX-M-1_, *bla*_SHV_**_,_** and *tet(A).* Another of the 18 isolates was genotypically ESBL-positive, carrying *bla*_SHV_ and co-carrying the carbapenemase gene *bla*_NDM_**.** It was classified as carbapenemase-positive**.** Six of the 18 strains were phenotypically classified as penicillinase-producing by VITEK2 based on their antimicrobial susceptibility profiles. Of these six, three did not harbor any acquired, plasmid-mediated antimicrobial resistance genes, which is consistent with the intrinsic resistance of *K. pneumoniae* to early penicillins via its chromosomal *bla*_SHV-1._ Of the remaining three penicillinase strains, one harbored acquired *bla_TEM_, bla*_SHV_, and *tet(A),* and the other two had only *bla*_SHV_ (Table 2S, Supplementary Material)_._ None of the *bla*_VIM_, *bla*_IMP_, or *bla*_KPC_ genes was detected.

In the non-fermenting group, *P. aeruginosa* isolates were classified as MDR (12%), XDR (35%), and pandrug resistant (5%) (Figure 4, Figure 5). The two metallo-β-lactamase genes detected in *P. aeruginosa* were *bla*_VIM_ and *bla*_NDM_ (2/17) (Figure 3), reflecting the carbapenem resistance profile.

**Figure 5:**
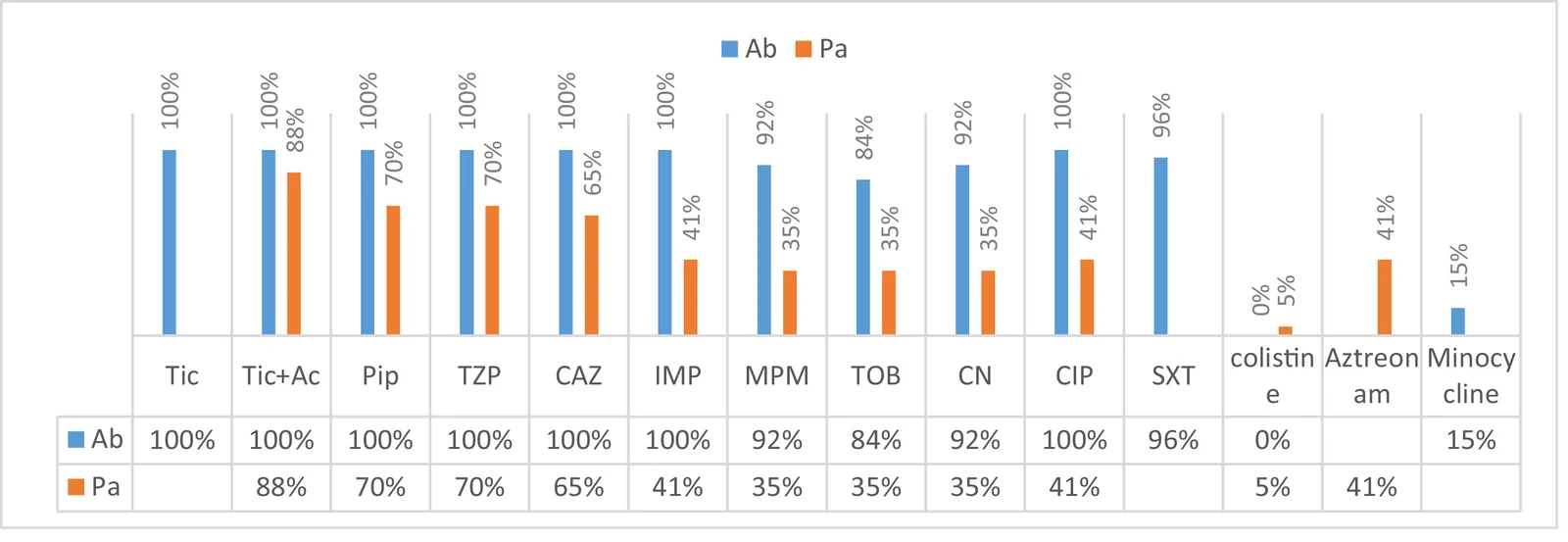
Resistance rate of non-fermenting bacteria. Each color on the bar corresponds to a bacterial type (Ab or Pa) and indicates the percentage of resistance to the antimicrobials listed in the table. Ab, *Acinetobacter baumannii*; CAZ, ceftazidime; CIP, ciprofloxacin; CN, gentamicin; IMP, imipenem; MPM, meropenem; Pa, *Pseudomonas aeruginosa*; Pip, piperacillin; SXT, cotrimoxazole; Tic, ticarcillin; Tic+Ac, Ticarcillin + acid clavulanic; TOB, tobramycin; TZP, piperacillin-tazobactam.

All *A. baumannii* isolates were susceptible to colistin according to the Unitary Minimal Inhibitory Concentration (UMIC) method. The majority (59%) had a reported colistin minimum inhibitory concentration (MIC) of 0.5 mg/l, while 8% had a reported MIC of 2 mg/l (Figure 1S, Supplementary Material).

The isolates' antimicrobial resistance rates are illustrated in Figure 5. All *A. baumannii* isolates were carbapenemase producers; the main β-lactamase detected was blaOXA-23, found in 13/26 isolates, while the remaining 13 strains did not harbor the *bla*_OXA-23_ gene (Figure 3). They were classified as MDR (19%) and XDR (81%) (Figure 4).

Only two *S. maltophilia* isolates were detected. The VITEK 2 analysis of resistance to cotrimoxazole showed that one isolate was resistant, while the other was intermediate.

### Molecular typing (PFGE and FT-IR)

#### PFGE results

All strains were successfully genotyped, revealing distinct pulsotypes. In particular, we discerned 18 distinct pulsotypes of *K. pneumoniae*, 14 pulsotypes of *P. aeruginosa*, six pulsotypes of *E. coli*, 17 pulsotypes of *A. baumannii*, four pulsotypes of *P. mirabilis*, and two pulsotypes of *S. maltophilia*. Dendrogram analysis excluded all outbreak strains because the pulsotypes were heterogeneous and showed no genetic relationship (Figure 2S; PFGE dendrograms, Supplementary Material).

#### FT-IR results

All strains were successfully genotyped, revealing different FT-IR profiles by isolate type. Specifically, we identified 10 distinct FT-IR profiles of *K. pneumoniae*, eight of *P. aeruginosa*, four of *E. coli*, 18 of *A. baumannii*, and four of *P. mirabilis* (Table 1; Tables 2S-5S and Figures 3S-7S in the Supplementary Material).

**Table 1:** Comparison of matching clusters: PFGE vs FT-IR.

Species	Number of PFGE clusters	Number of IR-biotyper clusters	Concordant clusters (%)
*Klebsiella pneumoniae*	*18*	*10*	*0 (0%)*
*Pseudomonas aeruginosa*	14	8	1/17 (6%)
*Escherichia coli*	6	4	4/7 (57%)
*Acinetobacter baumannii*	17	18	7/26 (27%)
*Proteus mirabilis*	4	4	4/4 (100%)

Species	Number of PFGE clusters	Number of IR-biotyper clusters	Concordant clusters (%)

*Klebsiella pneumoniae*	*18*	*10*	*0 (0%)*
*Pseudomonas aeruginosa*	14	8	1/17 (6%)
*Escherichia coli*	6	4	4/7 (57%)
*Acinetobacter baumannii*	17	18	7/26 (27%)
*Proteus mirabilis*	4	4	4/4 (100%)

Abbreviations: FT-IR, Fourier-transform infrared spectroscopy; PFGE, pulsed-field gel electrophoresis.

*S. maltophilia* was not typed because the PFGE pulsotypes showed no relationship between the two isolates.

#### Correlation results

The PFGE results revealed more clusters, while FT-IR identified fewer cluster profiles. Interestingly, the clustering patterns from both methods showed more differences than similarities. Regarding strain relationships, the dendrograms showed no relationship among isolates of all species analyzed by PFGE and IR-biotyper, even those from the same ICU during the same months. Also, all single strains not grouped into a cluster by PFGE across all species were not grouped by FT-IR either (Tables 2S-5S in the Supplementary Material).

#### Clustering concordance

Clustering concordance between PFGE and FT-IR was observed for *P. mirabilis* (adjusted Rand index = 1, Table 2).

**Table 2:** Clustering concordance between the PFGE and IR-biotyper genotyping techniques.

Species	Simpson's index of diversity for PFGE (95% CI)	Simpson's index of diversity for IR-biotyper (95% CI)	Adjusted Rand index (95% CI)
*Escherichia coli*	0.952 (0.855-1.000)	0.714 (0.394-1.000)	0.222
*Klebsiella pneumoniae*	1.000	0.908 (0.838-0.979)	0.000 (0.000-0.000)
*Acinetobacter baumannii*	0.926 (0.849-1.000)	0.692 (0.517-0.867)	0.304
*Pseudomonas aeruginosa*	0.978 (0.951-1.000)	0.882 (0.797-0.967)	0.180 (0.000-0.549)
*Proteus mirabilis*	0.900 (0.725-1.000)	0.900 (0.725-1.000)	1.000 (1.000-1.000)

Simpson's index of diversity is used to assess the discriminatory power of each typing method (PFGE and IR-biotyper). The typing method is perfectly discriminatory when the value is 1 or >0.9. In contrast, when the value is <0.5, the method has low discriminatory power. The adjusted Rand index is used to assess clustering concordance. Perfect concordance occurs when the value is 1; in contrast, when the value is near 0, there is no concordance.

Abbreviations: CI, confidence interval; IR, infrared; PFGE, pulsed-field gel electrophoresis.

## Discussion

### Antimicrobial resistance

In this study, a high level of resistance was observed in the ICU of a tertiary hospital in Rabat, Morocco, among bacteria isolated from patients with VAP. *A. baumannii* was the main non-fermenting Gram-negative bacterium. Conversely, an Egyptian study [4] found *K. pneumoniae* to be the most common causative microorganism (56%), followed by *P. aeruginosa* (13%) and *A. baumannii* (4%). A French multicentre study reported *E. coli* as the most dominant (22%), followed by *P. aeruginosa* (11%) and *Klebsiella* (8%), while no *A. baumannii* was detected [11]. In Italy, *A. baumannii* was the least detected non-fermenting bacterium (5.5%), while *P. aeruginosa* was the most frequently detected (22%), followed by *Klebsiella* (20.5%) [9]. Given the lower prevalence of Enterobacterales in our study, the antimicrobial profile remained clinically relevant. However, few studies have investigated their specific role in VAP. In our study, *P. mirabilis* was found in 6% of cases, similar to previous Moroccan studies. However, our results do not align with Karrati’s study [12], in which *P. mirabilis* was fully susceptible to imipenem and showed moderate resistance to ticarcillin and piperacillin (47.4%) and cefotaxime (5.3%). Conversely, our study aligns with a Tunisian study, in which *Proteus* isolated from ventilated patients was resistant to imipenem, penicillins, and broad-spectrum cephalosporins, including cefotaxime [13]. In that study, the *bla*_NDM_ gene was detected in 11 *P. mirabilis* isolates, of which 10/11 had previously been exposed to mechanical ventilation. In contrast, we found only one isolate harboring the *bla*_NDM_. This discrepancy is due to the intrinsic resistance to imipenem known in *Proteus* species [14], driven by lower affinity for penicillin-binding proteins and active efflux rather than acquired carbapenemase alone [14].

Our *E. coli* isolation rate (9.6%) was similar to those reported in the Egyptian study [6] and the South African study [15], which were 4.8% and 3.3%, respectively, but was lower than that reported in the French study [11], which was 34%. Fortunately, our isolates were still susceptible to carbapenems, which are recommended as empirical therapy for suspected VAP. Also, the prevalence of ESBL-producing *E. coli* is increasing; Bay et al. [16] found VAP related to ESBL-producing Enterobacterales to be 95%, and in our study, it was 71%.

*K. pneumoniae* isolates were the most dominant Enterobacterales in our study. They showed diverse resistance genes and harbored the highest number*.* This diversity explained the observed resistance phenotype. The most common mechanisms were carbapenemase activity, followed by ESBL activity. In Egypt, Ramadan et al. [17] reported that only *bla*_OXA-48_ and *bla*_KPC_ were found in *K. pneumoniae*. In contrast, our study detected no *bla*_KPC_ gene. Among the three species, *K. pneumoniae* was the most resistant of the Enterobacterales, exceeding *E. coli* and *P. mirabilis*. Comparing our results with earlier Moroccan studies*,* we noticed a concerning rise in antimicrobial resistance over time. For instance*,* a 2017 study by Mohammed et al. [18] reported a moderate rate of resistance to aminopenicillins and cephalosporins in *K. pneumoniae* (29%-37%). In our study, resistance rates were 55%-64%. This alarming spike was also observed for ciprofloxacin, rising from moderate rates below 50% to 70% across all three species.

Regarding the resistance profile of *A. baumannii,* we observed an alarming rise in ciprofloxacin resistance, from 97% in 2019 [12] to 100% in our study. In Uwingabiye et al.'s [19] study, all strains collected from Moroccan ICUs, including respiratory tract isolates, exhibited *bla*_OXA-23_-mediated carbapenem resistance. In comparison, our study confirms that *A. baumannii* in Morocco retains the *bla*_OXA-23_ gene, even though it was present in a lower proportion (50%) of our isolates. The high prevalence of *bla_OXA-23_* is compatible with global reports, which estimate it at 70%-100% [20]. Also, according to those previous studies, high levels of MDR and XDR were observed among *A. baumannii* isolates, which aligns with our research, where all isolates were susceptible to colistin. This aligns with a recent Moroccan study [21] but contrasts with Rongrungruang et al.'s [22] study, where *A. baumannii* was susceptible to colistin at only 52.3%. However, a moderate increase in the MIC values was observed (Figure 1S, Supplementary Material). Although the increase in MIC appears moderate, it remains concerning. Since colistin is considered the last-resort antibiotic in Morocco for treating infections caused by carbapenem-resistant *A. baumannii*, this trend signals a potential rise in resistance [23].

MDR *P. aeruginosa* is commonly isolated from patients with VAP. In this study, the resistance rate remained moderate compared with *A. baumannii* (Figure 3). The frequency of *P. aeruginosa* was 22%, in contrast to that in Jeyaraman et al.'s [24] article, in which

*P. aeruginosa* (49%) was the most commonly isolated VAP pathogen and showed 61% susceptibility to meropenem, which was similar to that in our study (65%). Resistance rates to other antimicrobials were moderate, except for ceftazidime. In comparison, polymyxin resistance was low at 7.7% in the *P. aeruginosa* VAP study. Regarding resistance classification, our results showed alarming rates, with isolates classified as MDR and XDR at 24% and 35%, respectively. This alarming *P. aeruginosa* XDR trend was explained by prophylactic antibiotic therapy, which triggers resistance selection among strains [25].

### Molecular genotyping

PFGE and FT-IR were used to analyze the genetic relationships among isolates of

*A. baumannii, K. pneumoniae, P. aeruginosa, E. coli, and P. mirabilis* from patients on mechanical ventilation in three Moroccan ICUs of a tertiary hospital. Specifically, PFGE pulsotype analysis showed significant heterogeneity among bacterial clones, indicating the absence of transmission of bacterial pathogens among hospitalized individuals. Furthermore, Papajk et al. [26] found that substantial clone variability supports the conclusion that VAP often originates from microaspiration of endogenous bacterial sources.

These results could help rule out an epidemiological outbreak.

FT-IR was used to monitor strain relationships in ICU cases of VAP. According to Cunha [27], the application of FT-IR in epidemiological outbreak detection was tested on 11 bacterial species, including *A. baumannii, K. pneumoniae, P. aeruginosa*, and *E. coli*, and, when compared with whole-genome sequencing, was found to be relevant. In our study, PFGE was the gold standard, and both methods confirmed that there was no relationship among the isolates.

However, in terms of clustering, FT-IR demonstrated the ability to classify certain strains within the same cluster despite being collected from disparate ICUs and over varying periods, whereas PFGE categorized them into distinct clusters. We observed this clustering difference among *K. pneumoniae, E. coli, P. aeruginosa,* and *A. baumannii*. This contrast might be due to the principle of strain typing, which varies between molecular (DNA-based analysis) and spectroscopic (carbohydrate-based analysis) techniques [28].

For *A. baumannii,* Guerrero-Lozano et al. [29] concluded that the IR-biotyper successfully enabled accurate clustering of *A. baumannii* isolates compared with PFGE. In our study, alignment occurred in only seven of 26 *A. baumannii* isolates. Conversely, Martak et al. [28] found that FT-IR performance was slightly lower for *A. baumannii* when assessing genomic relatedness using the IR-biotyper than with PFGE.

For *Pseudomonas*, our results align with those of Hu et al. [30]. The study confirms that PFGE classifies species with high resolution based on genetic typing, grouping many *Pseudomonas* strains into different types and resulting in clustering differences between IR-biotyper and PFGE. However, Martak et al. [28] found that FT-IR spectroscopy clusters clonal isolates with appropriate specificity but with lower sensitivity.

Regarding *P. mirabilis*, the IR-biotyper analysis grouped all the strains similarly to the PFGE groups. The clusters matched perfectly between the two methods. The PFGE results showed a high degree of consistency with the FT-IR data. This finding aligns with the review by Quintilas et al. [31], which highlighted that FT-IR has an 80% discriminatory power for *P. mirabilis.* However, few studies have evaluated the discriminatory capabilities of IR-biotyper for *P. mirabilis.*

### Study limitations

The limited number of VAP cases delayed prospective collection for 2 years. We also did not collect environmental bacteria, which could have enhanced the epidemiological analysis. Another limitation is that we did not use a reference method to confirm antimicrobial susceptibility; we used the VITEK 2 Compact System as the sole method, which further limited the analysis.

## Conclusion

*A. baumannii* was the predominant bacterium in VAP, while *Klebsiella spp.* were the most frequently isolated Enterobacterales. A high prevalence of MDR and XDR phenotypes was observed among *K. pneumoniae, while E. coli showed a high MDR profile. P. mirabilis, A. baumannii, and P. aeruginosa* caused VAP in patients in ICUs in a tertiary hospital in the capital of Morocco. The resistance was mainly due to the acquisition of resistance genes such as *bla*_CTX-M_, conferring resistance to third-generation cephalosporins, and *bla*_NDM_, *bla*_VIM_, and *bla*_OXA-23_, which confer carbapenem resistance. Moreover, epidemiological analysis revealed high heterogeneity among the different strains in each species.

## Funding

This research was funded by Pfizer Grant ID 52330333, by the BioMérieux/IM Alliance (Morocco), and by the Agreement between the Spanish Agency for International Development Cooperation and the Barcelona Institute for Global Health Private Foundation to support the Mediterranean Health Observatory based in Morocco (BOE-A-2020-5243). However, no specific funding was received to cover the costs of article processing and publication.

## Ethical approval

This study was conducted after obtaining approval from the Ethics Committee of the Faculty of Medicine and Pharmacy of Rabat (N° 68/16) and the National Commission for the Control of Personal Data Protection of the Kingdom of Morocco (N° A-RS-465/2019). The study adhered to the principles of the amended Declaration of Helsinki. Informed consent was obtained from the patients' legal representatives.

## Consent for publication

Not applicable.

## CRediT authorship contribution statement

**Sobha El Ftouh:** Writing – review & editing, Writing – original draft, Visualization, Validation, Resources, Project administration, Methodology, Investigation, Funding acquisition, Formal analysis, Data curation, Conceptualization. **Houssain Tligui:** Validation, Supervision, Resources, Project administration, Methodology, Investigation, Funding acquisition, Conceptualization. **Jordi Vila:** Writing – review & editing, Validation, Supervision, Resources, Methodology, Investigation, Conceptualization. **Larbi Aberouch:** Project administration, Investigation. **Tarek Dendane:** Supervision, Investigation, Data curation. **Latifa Oualili:** Investigation. **Oussama Ssouni:** Investigation. **Mamoun Faroudy:** Supervision. **Amine Ali Zeggwagh:** Conceptualization. **Mouhssine Doumiri:** Supervision, Investigation. **Cristina Pitart:** Visualization, Validation, Software, Methodology. **Maria Piquet:** Visualization, Validation, Supervision, Methodology, Conceptualization. **Elisabet Guiral:** Writing – review & editing, Visualization, Supervision, Project administration, Methodology. **Khalid Abidi:** Writing – review & editing, Validation, Supervision, Project administration, Methodology, Investigation, Funding acquisition, Conceptualization.

## Declaration of competing interest

The authors have no competing interests to declare.

## Appendix Supplementary materials

Image, application 1

Image, application 2

Image, application 3

Image, application 4

Image, application 5

Image, application 6

Image, application 7

Image, application 8

Image, image 9 dummy alt text

Image, image 10 dummy alt text

Image, image 11 dummy alt text

Image, image 12 dummy alt text

Image, image 13 dummy alt text
