# Supplementary material for: Microbiological profiles of Gram-negative bacteria isolated from respiratory samples of mechanically ventilated patients at a tertiary Moroccan hospital
Source: IJID Reg. 2026 Aug 11;21:100969. doi: 10.1016/j.ijregi.2026.100969 (PMC13627934; doi:10.1016/j.ijregi.2026.100969)
Supplement: Supplementary file 1 [file mmc1.docx]

**Caption Figure 1 :**

From 173 patients, only 78 MDR Gram-negative bacteria were double-checked by VITEK2 and MALDI-TOF and underwent genotyping. Based on PFGE genotyping results, 73 bacteria were genotyped a second time with the IR-biotyper and underwent antimicrobial resistance gene testing.
