# Supplementary material for: Microbiological profiles of Gram-negative bacteria isolated from respiratory samples of mechanically ventilated patients at a tertiary Moroccan hospital
Source: IJID Reg. 2026 Aug 11;21:100969. doi: 10.1016/j.ijregi.2026.100969 (PMC13627934; doi:10.1016/j.ijregi.2026.100969)
Supplement: Supplementary file 2 [file mmc2.docx]

NUMBERS AND PECENTAGE OF REPARTITION

| MIC mg/L | UMIC Ab |
| --- | --- |
| 0.125mg/L | 1 |
| 0.5 mg/L | 14 |
| 1mg/L | 7 |
| 2 mg/L | 2 |

**Figure 1S**. Inhibitory minimal concentration of colistin by the UMIC test for *A. baumannii*
