# Supplementary material for: Microbiological profiles of Gram-negative bacteria isolated from respiratory samples of mechanically ventilated patients at a tertiary Moroccan hospital
Source: IJID Reg. 2026 Aug 11;21:100969. doi: 10.1016/j.ijregi.2026.100969 (PMC13627934; doi:10.1016/j.ijregi.2026.100969)
Supplement: Supplementary file 3 [file mmc3.docx]

*Acinetobacter baumannii*

Figure 2S: PFGE dendrograms of *A.baumannii*, *P. aeruginosa*, *S. maltophilia*, *E. coli, K. pneumoniae, and Proteus mirabilis* recovered from cases of VAP patients in a tertiary hospital in Morocco
