# Supplementary material for: Microbiological profiles of Gram-negative bacteria isolated from respiratory samples of mechanically ventilated patients at a tertiary Moroccan hospital
Source: IJID Reg. 2026 Aug 11;21:100969. doi: 10.1016/j.ijregi.2026.100969 (PMC13627934; doi:10.1016/j.ijregi.2026.100969)
Supplement: Supplementary file 4 [file mmc4.docx]

| **Primers** | **Seq5'3'** | **Tm(ºC)** | **Base pairs (bp)** |
| --- | --- | --- | --- |
| NDM-1 F | CCAATATTATGCACCCGGTCG | 59,8 | 784 bp |
| NDM-1 R | ATGCGGGCCGTATGAGTGATTG | 62,1 | 784 bp |
| PCRm-OXA-48_F | ATG CGT GTA TTA GCC TTA TCG | 52,5 | 265 bp |
| PCRm-OXA-48_R | CAT CCT TAA CCA CGC CCA AAT C | 56,4 | 265 bp |
| PCRm-VIM_F | TGTCCGTGATGGTGATGAGT | 56 | 437 bp |
| PCRm-VIM_R | ATTCAGCCAGATCGGCATC | 55,2 | 437 bp |
| ctxm1-F | AAAAATCACTGCGCCAGTTC | 55,3 | 415 bp |
| ctxm1-R | AGCTTATTCATCGCCACGTT | 55,3 | 415 bp |
| ctxm2-F | CGACGCTACCCCTGCTATT | 58,8 | 552 bp |
| ctxm2-R | CCAGCGTCAGATTTTTCAGG | 57,3 | 552 bp |
| ctxm9-F | CAAAGAGAGTGCAACGGATG | 57,3 | 205 bp |
| ctxm9-R | ATTGGAAAGCGTTCATCACC | 55,3 | 205 bp |
| CTXM825_F | CGCTTTGCCATGTGCAGCACC | 52 | 307-308 bp |
| CTXM825_R | GCTCAGTACGATCGAGCC | 52 | 307-308 bp |
| Tem-F_multi | TCGCCGCATACACTATTCTCAGAATGA | 63,4 | 404 bp |
| Tem-R_multi | ACGCTCACCGGCTCCAGATTTAT | 62,4 | 404 bp |
| Shv-F_multi | ATGCGTTATATTCGCCTGTG | 55,3 | 900 bp |
| Shv-R_multi | TGCTTTGTTATTCGGGCCAA | 55,3 | 900 bp |
| tet(A)F | GCTACATCCTGCTTGCCTTC | 56.2 | 956 bp |
| tet(A)R | CATACATCGCCGTGAAGAGG | 55.5 | 956 bp |
| tet(B)F | TTGGTTAGGGGCAAGTTTTG | 55,3 | 415 bp |
| tet(B)R | GTAATGGGCCAATAACACCG | 57,3 | 415 bp |

**Table 1S:**

(a).Primer profile of *bla*_CTX-M_, *bla*_TEM_*, bla*_SHV_*, bla*_VIM_*, bla*_NDM_, tet (A), tet (B).

(b).Thermocycling conditions of every primer.

(a)

Tm**(ºC) :** annealing température ; C : Cytosine ; G : Guanine ; T : Thimine ; A :Adenine

| ***bla*_NDM_** | | |  | | ***bla*_OXA-48/VIM_** | |  |
| --- | --- | --- | --- | --- | --- | --- | --- |
| 1 cycle | 96ºC;3min | |  | | 1 cycle | 96ºC;3min |  |
| 30 cycles | 96ºC;30seg | |  | | 30 cycles | 96ºC;30seg |  |
|  | **61ºC;30seg** | |  | |  | **55ºC;30seg** |  |
|  | 72ºC;1min | |  | |  | 72ºC;30seg |  |
|  | 72ºC;10min | |  | |  | 72ºC;10min |  |
|  | 4ºC;∞ | |  | |  | 4ºC;∞ |  |
|  |  | |  | |  |  |  |
| ***bla*_CTX-M-1/CTX-M-9_** | | |  | | ***bla*_CTX-M-2_** | |  |
| 1 cycle | 96ºC;3min | |  | | 1 cycle | 96ºC;3min |  |
| 30 cycles | 96ºC;30seg | |  | | 30 cycles | 96ºC;30seg |  |
|  | **58ºC;30seg** | |  | |  | **58ºC;30seg** |  |
|  | 72ºC;30seg | |  | |  | 72ºC;1min |  |
|  | 72ºC;10min | |  | |  | 72ºC;10min |  |
|  | 4ºC;∞ | |  | |  | 4ºC;∞ |  |
|  |  | |  | |  |  |  |
| ***bla*_TEM/SHV_** | | |  | | **TET(A)/TET(B)** | |  |
| 1 cycles | 96ºC;3min | |  | | 1 cycle | 96ºC;3min |  |
| 30 cycles | 96ºC;30seg | |  | | 30 cycles | 96ºC;30seg |  |
|  | **58ºC;30seg** | |  | |  | **XºC;30seg** | Tet(A): **55.8°C**  Tet(B) : **56.3°C** |
|  | 72ºC;1 min | |  | |  | 72ºC; 1 min |  |
|  | 72ºC;10min | |  | |  | 72ºC;10min |  |
|  | 4ºC;∞ | |  | |  | 4ºC;∞ |  |
|  |  | |  | |  |  |  |
| ***bla*_CTX-M-8/CTX-M-25_** | | | |  |  |  |  |
| 1 cycle | | 96ºC;3min | |  |  |  |  |
| 30 cycles | | 96ºC;30seg | |  |  |  |  |
|  |  | **53ºC;30segg** | |  |  |  |  |
|  |  | 72ºC;30seg | |  |  |  |  |
|  |  | 72ºC;10min | |  |  |  |  |
|  |  | 4ºC;∞ | |  |  |  |  |

(b)
