# Supplementary material for: Microbiological profiles of Gram-negative bacteria isolated from respiratory samples of mechanically ventilated patients at a tertiary Moroccan hospital
Source: IJID Reg. 2026 Aug 11;21:100969. doi: 10.1016/j.ijregi.2026.100969 (PMC13627934; doi:10.1016/j.ijregi.2026.100969)
Supplement: Supplementary file 5 [file mmc5.docx]

| ID | Resistance genes | Sample collection period | ICUs | Pulsotypes  Cheff | FTIR biotyper profiles |
| --- | --- | --- | --- | --- | --- |
| vap 169 | *bla*_NDM_ + *bla*_CTXM1_ + *bla*_TEM_ + *bla*_SHV_ | 02/2024 | 2 | 1 | 2 |
| vap017 | *bla*_NDM_ + *bla*_SHV_ | 10/10/2022 | 2 | 2 | 10 |
| vap022 | *bla*_NDM_ + *bla*_CTXM1_ + *bla*_TEM_ + *bla*_SHV_ | 20/10/2022 | 2 | 3 | 1 |
| Vap038 | *bla*_NDM_ + *bla*_CTXM1_ + *bla*_TEM_ + *bla*_SHV_ | 27/12/2022 | 1 | 4 | 1 |
| Vap041 | *bla*_NDM_ + *bla*_CTXM1_ + *bla*_TEM_ + *bla*_SHV_ | 05/01/2023 | 1 | 5 | 2 |
| Vap045 | *bla*_CTXM1_ + tet (A) + *bla*_TEM_ + *bla*_SHV_ | 26/01/2023 | 1 | 6 | 6 |
| Vap049 | *bla*_OXA- 48-like_ + *bla*_NDM_ + *bla*_CTXM1_ + tet (A) + *bla*_SHV_ | 08/03/2023 | 1 | 7 | 8 |
| Vap060 | *bla*_SHV_ | 04/04/2023 | 3 | 8 | 2 |
| Vap074 | *bla*_CTXM1_ + tet (A) | 03/05/2023 | 1 | 9 | 7 |
| Vap088 | *bla*_CTXM1_ + *bla*_TEM_ | 08/06/2023 | 2 | 10 | 3 |
| Vap095 | *bla*_CTXM1_ + tet (A) + *bla*_TEM_ + *bla*_SHV_ | 21/06/2023 | 3 | 11 | 1 |
| Vap098 | *bla*_SHV_ | 26/06/2023 | 2 | 12 | 4 |
| Vap115 | tet (A) + *bla*_TEM_ + *bla*_SHV_ | 22/08/2023 | 2 | 13 | 9 |
| Vap118 | *bla*_OXA- 48-like_ + bla*_CTXM1_* + bla*_TEM_* + bla_SHV_ | 22/8/2023 | 1 | 14 | 2 |
| Vap120 | No resistance gene detected | 23/8/2023 | 1 | 15 | 5 |
| Vap123 |  | 4/9/2023 | 2 | 16 | 3 |
| Vap136 | *bla*_OXA- 48-like_ + *bla*_CTXM1_ + *bla*_TEM_ + *bla*_SHV_ | 13/10/2023 | 1 | 17 | 1 |
| Vap 154 |  | 26/12/2023 | 2 | 18 | 4 |

**Table 2 S. Information on *Klebsiella pneumoniae* isolates tested in this study**

ICUs : Intensive Care Units
