# Supplementary material for: Microbiological profiles of Gram-negative bacteria isolated from respiratory samples of mechanically ventilated patients at a tertiary Moroccan hospital
Source: IJID Reg. 2026 Aug 11;21:100969. doi: 10.1016/j.ijregi.2026.100969 (PMC13627934; doi:10.1016/j.ijregi.2026.100969)
Supplement: Supplementary file 6 [file mmc6.docx]

**Table 3 S: Information on *Acinetobacter baumannii* isolates tested in this study.**

| ID | Resistance genes | Sample collection period | ICUs | Pulsotypes Cheff | FTIR biotyper Profiles |
| --- | --- | --- | --- | --- | --- |
| Vap007 | *bla*_OXA-23_ | 18/07/2022 | 1 | 2 | 4 |
| Vap004 | *bla*_OXA-23_ | 7/7/2022 | 3 | 15 | 18 |
| Vap24 | *bla*_OXA-23_ | 3/10/2022 | 1 | 3 | 4 |
| Vap029 | Negative | 22/11/2022 | 3 | 16 | 1 |
| Vap032 | *bla*_OXA-23_ | 2/12/2022 | 1 | 4 | 3 |
| Vap040 | *bla*_OXA-23_ | 28/12/2022 | 1 | 17 | 7 |
| Vap057 | *bla*_OXA-23_ | 3/3/2023 | 1 | 3 | 4 |
| Vap063 |  | 13/04/2023 | 3 | 4 | 3 |
| Vap075 | *bla*_OXA-23_ | 8/5/2022 | 1 | 5 | 1 |
| Vap077 | Negative | 11/05/2023 | 1 | 6 | 1 |
| Vap080 | Negative | 13/5/2023 | 2 | 7 | 1 |
| Vap089 | Negative | 9/6/2023 | 3 | 8 | 1 |
| Vap093 | Negative | 14/6/2023 | 2 | 9 | 1 |
| Vap095 | Negative | 21/6/2023 | 3 | 1 | 1 |
| Vap105 | *bla*_OXA-23_ | 6/7/2023 | 1 | 10 | 2 |
| Vap107 | *bla*_OXA-23_ | 13/7/2023 | 3 | 11 | 5 |
| Vap112 | Negative | 15/8/2023 | 1 | 1 | 1 |
| Vap 116 | Negative | 22/08/2023 | 2 | 1 | 1 |
| Vap119 | Negative | 22/08/2023 | 1 | 1 | 1 |
| Vap120 | Negative | 23/8/2023 | 1 | 1 | 1 |
| Vap128 | *bla*_OXA-23_ | 14/9/2023 | 3 | 12 | 6 |
| Vap130 | Negative | 25/9/2023 | 2 | 13 | 1 |
| Vap142 | *bla*_OXA-23_ | 30/10/2023 | 1 | 14 | 3 |
| Vap144 | Negative | 10/11/2023 | 1 | 1 | 1 |
| Vap 149 | *bla*_OXA-23_ | 18/12/2023 | 1 | 2 | 4 |
| Vap165 | *bla*_OXA-23_ | 25/1/24 | 1 | 1 | 1 |

ICUs : Intensive care units
