# Supplementary material for: Microbiological profiles of Gram-negative bacteria isolated from respiratory samples of mechanically ventilated patients at a tertiary Moroccan hospital
Source: IJID Reg. 2026 Aug 11;21:100969. doi: 10.1016/j.ijregi.2026.100969 (PMC13627934; doi:10.1016/j.ijregi.2026.100969)
Supplement: Supplementary file 7 [file mmc7.docx]

| **ID** | **Resistance genes** | **Sample collection period** | **ICUs** | **Pulsotype Cheff** | **FTIR biotyper profiles** |
| --- | --- | --- | --- | --- | --- |
| Vap011 | *bla*_NDM_ | 19/09/22 | 1 | 4 | 1 |
| Vap019 | Negative | 12/10/2022 | 2 | 7 | 4 |
| Vap037 | Negative | 20/12/2022 | 1 | 11 | 7 |
| Vap064 | Negative | 14/4/2023 | 3 | 1 | 2 |
| Vap067 | *bla*_NDM_ | 18/04/2023 | 3 | 5 | 3 |
| Vap080 | Negative | 13/5/2023 | 2 | 10 | 5 |
| Vap083 | *bla*_VIM_ | 01/06/2023 | 3 | 3 | 3 |
| Vap086 | Negative | 5/6/2023 | 2 | 13 | 5 |
| Vap088 | Negative | 8/6/2023 | 2 | 6 | 1 |
| Vap090 | Negative | 12/6/2023 | 3 | 1 | 2 |
| Vap094 | Negative | 14/6/2023 | 1 | 14 | 6 |
| Vap112 | Negative | 15/8/2023 | 1 | 9 | 8 |
| Vap124 | Negative | 4/9/2023 | 2 | 2 | 1 |
| Vap128 | *bla*_VIM_ | 14/9/2023 | 3 | 3 | 2 |
| Vap130 | Negative | 25/9/2023 | 2 | 2 | 1 |
| Vap135 | Negative | 6/10/2023 | 2 | 12 | 1 |
| Vap136 | Negative | 13/10/2023 | 1 | 8 | 4 |

**Table 4 S.** **Information on *Pseudomonas aeruginosa* isolates tested in this study**

ICUs : Intensive Care Units
