# Supplementary material for: Microbiological profiles of Gram-negative bacteria isolated from respiratory samples of mechanically ventilated patients at a tertiary Moroccan hospital
Source: IJID Reg. 2026 Aug 11;21:100969. doi: 10.1016/j.ijregi.2026.100969 (PMC13627934; doi:10.1016/j.ijregi.2026.100969)
Supplement: Supplementary file 8 [file mmc8.docx]

**Table 5 S: Information on *E. coli* isolates tested in this study**

| ID | Resistance genes | Sample collection period | ICUs | Pulsotypes Cheff | FTIR biotyper profiles |
| --- | --- | --- | --- | --- | --- |
| Vap035 | *bla*_CTXM1_ | 12/12/2022 | 1 | 2 | 1 |
| Vap039 | *bla*_CTXM1_ | 27/12/2022 | 3 | 1 | 1 |
| Vap042 | *bla*_OXA-48like_ + *bla*_NDM_ + *bla*_TEM_ | 18/01/2023 | 1 | 6 | 2 |
| Vap044 | *bla*_CTXM1_ | 25/01/2023 | 1 | 1 | 1 |
| Vap130 | *bla*_CTXM1_ | 25/9/2023 | 2 | 4 | 4 |
| Vap135 | *bla*_TEM_ | 6/10/2023 | 2 | 3 | 3 |
| VAP153 | *bla*_CTXM1_ | 26/12/2023 | 1 | 5 | 1 |

ICUs : Intensive Care Units
